# Anatomy Camp: A Medical Student-Run Outreach Program

**DOI:** 10.1007/s40670-024-01991-4

**Published:** 2024-01-29

**Authors:** Ethan P. Winter, Josika Sammarco, Vivian Hua, Orlando M. Martinez, Tianqi Xiao, Susanne Wish-Baratz

**Affiliations:** 1 Case Western Reserve University School of Medicine, Cleveland, OH USA; 2 Department of Anatomy, School of Medicine, Case Western Reserve University, 10900 Euclid Avenue, Cleveland, OH 44106 USA

**Keywords:** Anatomy, Community health diversity, K-12 outreach programs

## Abstract

Anatomy Camp is a medical student-run program for underserved youth from communities near the university. Middle and high school students are invited to visit the medical school for an afternoon of interacting with medical students, informal learning of anatomy and wellness, and becoming inspired to consider medical and STEM professions.

For over a decade, Case Western Reserve University School of Medicine (SOM) has been hosting “Anatomy Camp.” Anatomy Camp is a medical student-run activity aimed to inspire the next generation of potential physicians and healthcare professionals with a focus on underserved youth. Underserved youth is defined as adolescents who have inequitable access to educational resources [1]. These students often lack specific career knowledge and skills necessary to form postsecondary plans, have limited access to career preparation experiences, and lack trusted adult mentors in diverse careers [1]. Many of these students are also underrepresented in medicine (UIM). Research shows that a diverse physician workforce that reflects the population it serves is associated with better health outcomes [2]. Despite efforts to increase UIM applicants and matriculants to medical school, matriculation rates have not changed in decades [2]. This trend is also observed more broadly across the entire science, technology, engineering, and math (STEM) education sector, despite the fact that STEM skills have become increasingly important in the twenty-first century economy [3]. These students may initially believe that medical and STEM careers are unattainable for them. Thus, the core mission of Anatomy Camp is to expose, inspire, and guide students to the understanding that these careers are achievable and highly rewarding.

Anatomy Camp invites classes comprised of underserved youth from local middle and high schools to visit the SOM and learn about topic-specific human anatomy and age-appropriate wellness themes as well as the requirements for pursuing medical and STEM careers. Anatomy Camp runs for approximately 3 hours and is scheduled as an afternoon fieldtrip. The session begins with an introduction, during which medical student volunteers introduce themselves and provide a brief presentation that includes the day’s agenda. During the main portion of Anatomy Camp, groups of approximately ten students rotate through four stations (Fig. 1). One or two medical students serve as guides to a group for the entire visit, providing important continuity and building rapport with the students. Each station is staffed by two medical student instructors. Though the attendees themselves take an active role in each station, medical student leaders provide important mentorship and structure to the sessions and facilitate questions that arise. Clinical case stations are abbreviated and simplified from those used in the medical school’s curriculum. Content is variable, though common topics that have been used include cardiology, pulmonology, and musculoskeletal anatomy. When relevant, the visiting youth use mixed reality devices to view the pertinent anatomy in 3D. Anatomy is a particularly engaging subject for the students due to its relatable nature.

**Fig. 1:**
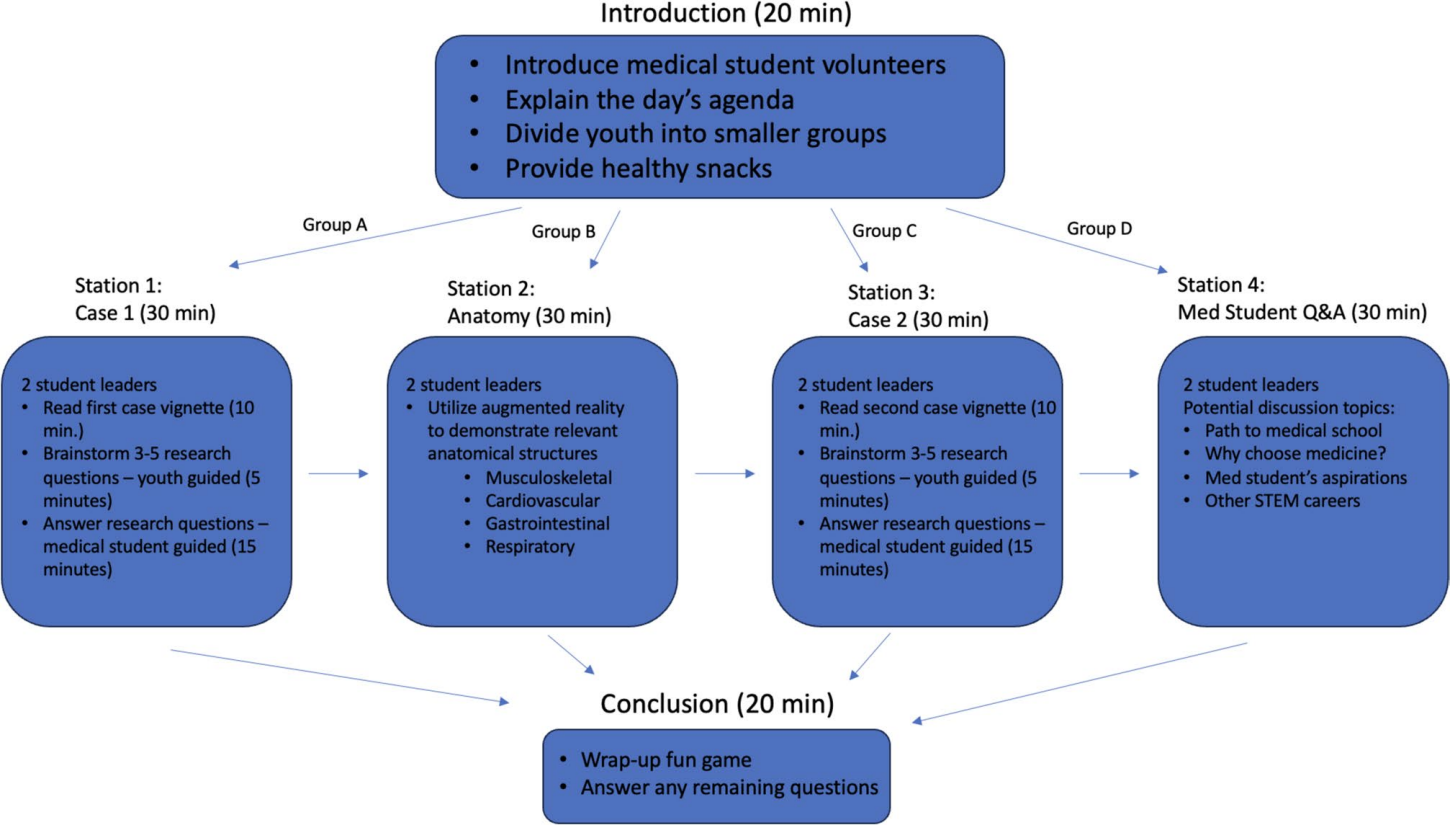
Anatomy Camp lesson plan

One station is typically dedicated to questions and answers. This provides attendees an opportunity to ask medical students questions about their lives and paths to medical school in an informal setting. After rotating through each station, the attendees all reconvene to wrap up the session and play a game that reinforces the content of the camp. Incidental feedback from visiting students repeatedly indicates that after an afternoon of Anatomy Camp, students learned, enjoyed, and desired to return for a future visit.

Behind the scenes, approximately five medical students take the lead, serving as the main organizers of the program. They begin halfway through their first year and serve for 1 year, handing off roles before their dedicated study period for STEP 1 in the spring of second year. Leadership duties include recruiting local schools, organizing volunteers, and developing curriculum. Medical student volunteers benefit greatly from participating in Anatomy Camp, and many choose to volunteer multiple times. Communication and teaching skills are an important part of the medical profession, and Anatomy Camp provides students with an opportunity to practice these skills in a low-stress environment. Anecdotal feedback from volunteers indicates that this is a fulfilling opportunity through which they feel they make a positive impact on the local community.

Anatomy Camp can be especially rewarding for volunteers early in their medical school careers as it provides an opportunity to interact with the youth in the communities surrounding their university. It is a unique opportunity for medical students to make a positive impact on the region and inspire the next generation of medical and STEM professionals. The model presented herein can easily be generalized to programs throughout the country.
